# Prevalence and psychosocial predictors of cyberaggression and cybervictimization in adolescents: A Spain-Ecuador transcultural study on cyberbullying

**DOI:** 10.1371/journal.pone.0241288

**Published:** 2020-11-11

**Authors:** Antonio J. Rodríguez-Hidalgo, Oswaldo Mero, Eva Solera, Mauricio Herrera-López, Juan Calmaestra

**Affiliations:** 1 Department of Psychology, Universidad de Córdoba, Córdoba, Spain; 2 Management, Development and Executive Secretariat Faculty, University Laica Eloy Alfaro, Manabí, Ecuador; 3 International University of La Rioja, Logroño, Spain; 4 Department of Psychology, University of Nariño, San Juan de Pasto, Colombia; Temple University, UNITED STATES

## Abstract

The present study aims to collect data about the prevalence of cyberbullying and the role of self-esteem, empathy, and social skills in predicting cybervictimization and cyberaggression in two different countries: Spain and Ecuador. Additionally, it compares the similarities found in both countries. A wide sample of adolescents from Secondary Education (*N =* 24943; mean age = 13.92; SD = 1.30, girls = 49.9%) from both countries (Spain = 14,206 and Ecuador = 10,737) took part by filling in a set of self-reports. Weighted analyses and structural equation models were used. The results revealed that 8.8% were cybervictims, 3.1% were cyberaggressors and 4.9% cybervictims-cyberaggressors in Spain; whereas 8.7% were cybervictims, 5.1% were cyberaggressors and 14.3% were cybervictims-cyberaggressors in Ecuador. Cybervictimization could be predicted in both countries by means of self-deprecation and social skills, although the meaning of some skills was different depending on the country. Cyberaggression could be predicted in both countries by means of empathy, assertiveness, and conflict-resolution skills, as well as by communicative and relational skills. Self-deprecation was a predictor of cyberaggression only in Spain. These results are discussed, and educational inferences are drawn for prevention.

## Introduction

Adolescents spend most of their time in high school, where they are involved in social activities with their classmates and teachers [1]. In recent years, this social dynamic has been extended and redefined by means of the use of digital relationships. More and more adolescents interact in the cyberspace, exceeding the relational limits that physical space has regarding time, space and the number of people connected [2]. Adolescents' accessibility to internet via mobile devices, technological advances and the rise of cyber-relationships contribute to the increased risk of being involved in cyberbullying [3, 4]. The scientific literature on the psychosocial factors related to this phenomenon is increasing. Nevertheless, three out of four of the studies on cyberbullying have been carried out in European countries or the USA [5]. This phenomenon has hardly been studied in countries of other more disadvantaged regions, such as the South American countries [6–8]. However, there is a small number of cross-cultural studies. Therefore, there is a need to deepen this line, through cross-cultural studies between countries of North America and Europe with countries in South America, which can contribute to: a) identify stable psychosocial factors in various contexts; and b) know the psychosocial factors that have a unique behavior in different countries. This would make possible to expand the evidence-based knowledge to design more effective prevention strategies and/or programs at a global level, and to contemplate specific strategies and actions adapted to the peculiarities of each cultural context. Additionally, this knowledge could make prevention programs more effective.

### Cyberbullying

Cyberbullying is an interpersonal, aggressive, repeated behavior whose purpose is harming a victim by means of information and communication technologies [9–13]. The cyberaggression that causes cybervictimization -the two dimensions of cyberbullying- is developed by means of cyberspace and other telematic channels, so it can be produced and reproduced in any place and at any time [7]. For cyberbullying to happen, it takes at least two people. The cybervictim who receives the cyberaggression, and therefore is cybervictimized, and the cyberaggressor who commits it.

Cyberbullying is associated with some psychological, social, educational, health and family problems that extend to those involved in the phenomena [3, 14–18]. However, at a greater extent, cybervictims may suffer discrimination among peers, depression, aggressiveness, anxiety, fear, somatic disorders, deterioration of self-esteem, academic problems, substance use, and suicidal attempts and ideation [15, 17, 19, 20].

Being a cybervictim increases the risk of becoming a cyberaggressor [21, 22]. Some studies have shown that becoming a cybervictim or cyberaggressor are not separate processes, but they even share quite a few risk factors [23]. Sometimes, there are people who are victims and aggressors at the same time, the so-called aggressor/victim in traditional bullying [24] and cyberbully/cybervictim in cyberbullying situations. High levels of antisocial behavior seem to be related to high levels of involvement as a cybervictim, cyberaggressor and cyberbullying observer [19, 25]. To prevent adolescents' involvement in this phenomenon, and the possible vicious circle between cybervictimization and cyberaggression, it is necessary to study its prevalence and the potential associated factors.

Studies on cyberbullying carried out in European and North American countries have contributed to generating knowledge for its prevention in these places. In these regions, studies about the prevalence of cyberbullying, regarding the different involvement roles, offer disparate data. This is partially due to the fact that the instruments of measurement used in the studies are different in many cases. For example, in the USA, Lee et al. [26] indicated that, considering a wide sample of adolescents, they observed that 13.6% were directly involved in cyberbullying: 5.1% were cybervictims; 3.9% were cyberaggressors and 4.6% were cybervictims-cyberaggressors. A systematic review of studies conducted in the USA with different measurement instruments shows that: between 1% and 41% are cyberbullies; between 3% and 72% are cybervictims; and between 2.3% and 16.7% are cyberbullying victims [27]. In Spain, specifically in Andalusia, it was observed that 31.2% of adolescents admitted being directly involved in bullying: 9.3% as cybervictims, 5.5% as cyberaggressors and 3.4% as cybervictims-cyberaggressors [28]. Also, in Spain, Larrañaga, Yubero, Ovejero, & Navarro's study [29] on adolescents showed that 26.6% were cybervictims, of whom 22% had become cybervictims promptly and 6.6% were serious cybervictims, without differences regarding sex. In recent years, the number of studies carried out on adolescents from Latin America has increased; but most of them are not published in impact journals [30]. There are countries where very few studies with scientific rigor and large samples have been carried out, as is the case of Ecuador. In this country, Pieschl, Kuhlmann, & Porsch [31] observed that 55% adolescents recognized themselves as cybervictims while 52% were cyberaggressors. This study shows that the levels of cyberbullying in Ecuador are higher than in Spain but, as pointed out in other works, the dynamics of the phenomenon is similar [32].

Recently, transnational studies are being carried out between European countries and Latin American countries. For example, Spain-Colombia transnational study on adolescents carried out by Herrera-López, Casas, Romera, Ortega-Ruiz, & Del Rey [33] showed that in Spain (Andalusia), 9.3% were cybervictims, 5.3% were cyberaggressors and 6.4% were cybervictims-cyberaggressors; whereas in Colombia, 10.7% were cybervictims, 2.5% were cyberaggressors and 5.5% were cybervictims-cyberaggressors [34]. This emerging line of transnational studies between favored and disadvantaged regions, by using similar instruments and methodology, could provide knowledge of great strategic value for the prevention of cyberbullying. In view of the scarce scientific literature on cyberbullying in Ecuador, the comparative study of this phenomenon with a European country with which it has strong historical-cultural ties, such as Spain, could be of great interest.

The study of individual factors related to cyberbullying has provided elements for the understanding of this phenomenon [35]. It seems especially relevant to continue deepening the study of individual factors related to the psychosocial life of adolescents. The immediate psychosocial dynamics, or immediate context, can be an aggravating factor and provide clues to understand cyberbullying [36, 37]. For these reasons, it is relevant to review the role of self-esteem, empathy, and social skills in relation to cyberbullying.

### Cyberbullying and self-esteem

Self-esteem is a feeling towards oneself depending on its characteristics and can be positive or negative [38]. In the last years, the number of studies based on the relation between cybervictimization and self-esteem in adolescents has increased [39–42]. Additionally, it has been observed that cybervictimization has a negative connection with self-esteem [39, 40, 43]. Cybervictims show lower levels of self-esteem, sense of belonging to school and commitment with it [42], fact that is more accentuated in girls than in boys [17]. Some studies have revealed that low self-esteem is a predictor of cybervictimization [40, 44, 45] and emphasized the fact that high self-esteem is a protective factor for cybervictimization.

In their studies, Bayraktar et al. [43] and Garaigordobil [46] suggested a negative correlation between cyberaggression and self-esteem; however, in the first study, it was shown that those who showed a lower self-esteem tended to be cyberaggressors/cybervictims while the second study indicated that they were pure cyberaggressors. On the other hand, Bergmann & Baier's study [21] slightly relates high self-esteem to a greater risk of cyberaggression: the prevalence of cyberaggression was correlated to being a girl, having high self-esteem, low empathy, low marks at school or being a bully in the school, among other issues. Additionally, some studies have revealed that a low level of self-esteem predicts cyberaggression [44, 45].

To summarize, most studies indicate that self-esteem is negatively correlated with cybervictimization. A considerable section of the studies also coincides in pointing out that self-esteem is negatively correlated with cyberaggression.

### Cyberbullying and empathy

Empathy could be defined as the personal ability to register and be in solidarity with others’ feelings [47, 48]. The levels of an aid behavior towards the others are positively associated with the levels of affective empathy [49]. Cognitive empathy is positively associated with aid behaviors towards cybervictims [50]. Nevertheless, low levels of empathy and moral connection are associated with higher levels of cyberaggression [21, 46]. Both cognitive and affective empathy are negatively associated with cyberaggression [51–54]. For some studies, the strongest association occurs with affective empathy [51] and for others, with cognitive empathy [52]. The systematic review and metanalysis on 25 studies by Zych, Baldry, Farrington, and Llorent [55] conclude that high levels of cyberaggression are related to low levels of empathy; and that cybervictims usually present higher levels of affective empathy.

Hence, it was observed that empathy helps to predict cyberaggression and cybervictimization [44]. Affective and cognitive empathy predict cyberaggression regardless sex, age, and nationality [52]. However, a short-term longitudinal study with a sample of less than half a thousand of Greek adolescents concluded that neither empathy nor sex were predictors of cyberaggression or cybervictimization [56]. On the other hand, in young adolescents, it has been observed that neither affective empathy nor cognitive empathy were predictors of cyberaggression [57].

The literature points out that empathy is positively related to cybervictimization. However, the reviewed works suggest that empathy is negatively related to cyberaggression.

### Cyberbullying and social skills

Social skills are cognitive routines or behaviors that allow to establish or maintain positive relationships with others [58, 59]. The review of the scientific literature shows that the educational prevention of cyberbullying in adolescents at a global level has as one of its main lines of actions the promotion and improvement of social skills [60–63]. In the last years, the study of relations between social skills and the involvement in cyberbullying has increased [7, 64]. More and more studies support the association of interpersonal difficulties with the victimization of cybernetics bullying during adolescence [65].

In the USA and Australia, Hemphill & Heerde [66] carried out a study where they concluded that presenting deficient social skills and a low social competence were associated with being a cybervictim. Furthermore, Navarro Yubero, Larrañaga, & Martínez [67] carried out a study with a small sample of Spanish adolescents where they observed that social anxiety, communicative difficulties with peers and the lack of social skills were predictors of cybervictimization. In a study also carried out in Spain with a wider sample of adolescents [7], it was observed that communicative social or relational skills, conflict-resolution skills and assertiveness did not show a predictive power of cybervictimization in none of the ethnic-cultural groups controlled by country of birth (Spain, Morocco, Romania, Colombia and Ecuador). Nevertheless, some social skills showed a predictive ability on cyberaggression for some ethnic-cultural groups: low assertiveness was a predictor of cyberaggression in those who were born in Spain; and communicative skills acted as a positive predictor of cyberaggression for those who came from Colombia and Spain. Garaigordobil pointed out that, in Spain, adolescents involved in cyberbullying used aggressive behavior more as conflict resolution techniques than those not involved [25].

Studies show that social skills appear to be related to cybervictimization as well as cyberaggression. However, regarding the meaning of these relationships—whether they are positive or negative—there are no conclusive results.

### The present study

According to the scientific literature, cyberbullying has a different prevalence across different regions and countries. In general, it has been observed that this phenomenon is usually related to self-esteem, empathy, and social skills. To prevent cyberbullying, it is necessary to know more about the prevalence of this phenomenon and the predictors of cyberaggression and cybervictimization on wide significant samples from different cultural contexts by means of instruments approved and validated internationally [7]. In the same line, it is important to examine how generalizable the phenomenon is in different context. The literature review shows that the emerging line of transnational studies between European and Latin American countries, although it is incipient, is beginning to offer significant contributions to alleviate cyberbullying in disadvantaged countries. There are very few comparative studies between Spain and Ecuador in this regard. For this reason, this transnational study focuses on these two countries. However, the study, based on the results obtained, has provided: a) general proposals for the prevention of cyberbullying; and b) country-specific prevention proposals, which can increase their effectiveness in each context [32]. For this reason, a transnational study has been developed with school-going adolescents in Spain and Ecuador regarding the following objectives:

- To study the prevalence of cyberbullying in adolescents considering the involvement roles in Spain and Ecuador.
- To know the predictive capacity of some psychosocial variables—self-esteem, empathy, and social skills—on cyberaggression and cybervictimization.
- To compare the predictors of cyberaggression and cybervictimization in adolescents from Spain and Ecuador.

The following hypotheses have been studied:

1. Self-esteem will be negatively associated with cybervictimization and cyberaggression.
2. Empathy will be positively associated with cybervictimization and negatively associated with cyberaggression.
3. Social skills will be associated with cybervictimization and cyberaggression.

## Materials and methods

### Participants

In total, 33,303 subjects filled in the data collection instruments. Nevertheless, 8,360 participants did not actually fill in any item of the instruments. That is why, the total sample for this study is formed by 24,943 students whose ages ranged from 11 to 18 (mean age = 13.92; SD = 1.30), of whom 49.9% were girls. The sample was collected in two countries: Spain (n = 14206; mean age_Spain_ = 14.03; SD = 1.39; girls = 50.7%) and Ecuador (n = 10737; mean age_Ecuador_ = 13.77; SD = 1.17; girls = 48.9%). The Spanish sample was collected by means of a conglomerate random sampling in the national territory, while the Ecuadorian sample was obtained following the same methodology but only in the geographic region 4 of Ecuador that consists of the wide provinces of Manabí and Santo Domingo de Los Tsáchilas.

### Instruments

We used a set of self-reports formed by 4 different questionnaires. Some questions regarding socio-demographic data were included in the set.

In order to measure cyberbullying, the Spanish version of the *European Cyberbullying Intervention Project Questionnaire*—ECIP-Q—was used [68]. The questionnaire was composed of two dimensions: cybervictimization and cyberaggression. It includes a total of 22 items—11 items per dimension—and it is answered by means of a 5-point Likert-type scale (0 = never; 1 = Yes, once or twice; 2 = Yes, once or twice a month; 3 = Yes, once a week; 4 = Yes, more than once a week). A sample item from the cybervictimization dimension is *“Someone said nasty things to me or called me names using texts or online messages”* and from the cyberaggression dimension is *“I spread rumors about someone on the Internet”*. The reliability values for this study are optimal (α_cybervictimization_ = 0.896; α_cyberaggression_ = 0.918). The factorial structure is validated with samples from Spain and Colombia that showed good results [33, 68]. Additionally, optimal values are also obtained in its factorial structure [69]: Satorra Bentler *χ*^2^_S-B_^=^ 20278.528, *p* < 0.001, NNFI = 0.982, CFI = 0.981, IFI = 0.983, RMSEA = 0.062 (90% CI: 0.061–0.063). In order to establish the different involvement roles—not involved; cybervictim, cyberaggressor and cybervictim-cyberaggressor—, the correction of the instrument proposed by the authors was used [11]. Cybervictims scores equal or higher than 2 (*Yes*, *once or twice a month*) in any of the items of cybervictimization and scores equal or lower that 1 (*Yes*, *once or twice*) in all the items of cyberaggression. Cyberaggressors scores equal or higher than 2 (*Yes*, *once or twice a month*) in any of the items of cyberaggression and scores equal or lower that 1 (*Yes*, *once or twice*) in all the items of cybervictimization. Cybervictim-cyberaggressor scores in any of the items of both cyberaggression and cybervictimization with a score equal or higher than 2 (*Yes*, *once or twice a month*).

Moreover, the Rosenberg Self-Esteem Scale—RSES—questionnaire was used to measure self-esteem [38]; this questionnaire was adapted and validated by Martín-Albo, Núñez, Navarro and Grijalvo [70] and Viejo [71]. This instrument can be used as one-factor instrument with a general value for self-esteem; or as a two-factor instrument, with two dimensions: self-confidence (five items positively formulated) and self-deprecation (five items negatively formulated). An example of an item for self-confidence is *“On the whole*, *I am satisfied with myself”* while *“I feel I do not have much to be proud of”* is an example of a self-deprecation item. The two-dimension model matched with the data collected for this research. The values of the scale of measurement range from 1 to 4. The reliability analyses have shown quite a few indexes (α_self-confidence_ = 0.776; α_self-deprecation_ = 0.757). The CFA showed optimal levels of the instrument (χ^2^_S-B_ = 2385.949, *p* < 0.001; CFI = 0.981; NNFI = 0.972; RMSEA = 0.053 [90% CI: 0.051–0.054]).

The Basic Empathy Scale—BES—[48] was used in a version adapted by Oliva et al. [59] to measure the levels of empathy. The scale is composed of 9 items (e.g. *“After being with a friend who is sad about something*, *I usually feel sad”*). The one-factor model that was used for this study shows optimal reliability values (α_empathy_ = 0.914). The CFA showed optimal levels for the factor structure of the instrument (χ^2^_S-B_ = 2892.841, *p* < 0.001; CFI = 0.973; NNFI = 0.963; RMSEA = 0.064 [90% CI: 0.063–0.067]). The scale of measurement of each item ranges from 1 (*Totally disagree*) to 5 (*Totally agree*).

The Social Skills Scale was used to measure the levels of social skills [59]. This scale is composed of 12 items with values ranging from 1 (*Totally false*) to 7 (*Totally true*). This questionnaire is subdivided in 3 dimensions. The first of them is called “Communicative or relational skills” and it is composed of 5 items (α_communicative_social_ = 0.793) (e.g. *“I have a hard time starting a conversation with someone I don't know”*). This dimension refers to the degree to which adolescents perceive that they are capable or not at communicating and relating to people [59]. The second dimension is “Assertiveness”, composed of 3 items (α_assertiveness_ = 0.747) (e.g. *“I usually praise or congratulate my classmates when they do something well”*). Assertiveness refers to the perception that adolescents have about their ability to be assertive, that is, to express their own ideas or request information in an appropriate way and without being aggressive [59]. Finally, the third dimension, “Conflict-resolution skills”, is composed of 4 items (α_conflict-resolution_ = 0.805) (e.g. *“When I have a problem with another boy or girl*, *I put myself in their shoes and try to fix it”*). This dimension refers to the adolescent's perceived ability to resolve conflictive interpersonal situations in which they can act to find solutions. The CFA showed optimal levels for the three-factor structure of the instrument (χ^2^_S-B_ = 3953.279, p = 0.001; CFI = 0.984; NNFI = 0.982; RMSEA = 0.055 [90% CI: .054-.057]).

### Procedure

A similar procedure was followed in both countries. The researchers got in contact with the different educational centers—previously selected in a random way—and asked their management teams for cooperation. Once the data collection was approved by the center, an informed consent letter was delivered to the students’ family. Written informed consent was obtained from the parents/guardians via the schools. The only difference between both countries was due to the unequal presence of Information and Communication Technologies in their schools. In Spain the collection was online, while in Ecuador it was by means of questionnaires in paper format due to the poor availability of technological devices and the lack of access to internet in their schools. In both countries the same standard was used in data collection. The interviewers were trained through an information sheet and a written collection protocol. The interviewer solved all the doubts that the respondents had. In both countries, a class session was used for data collection. Anonymity in responses was preserved. Additionally, the participation to fill in the questionnaire was voluntary, and students were informed that they could revoke their consent at any time. No relevant incidences were detected when collecting data. The methodology followed the ethical principles according to the Declaration of Helsinki. The procedure was approved by the Ethics Committee of the University of Córdoba and the research was conducted in line with national and international ethical standards.

### Data analysis

The following statistical programs were used to analyze the collected data: SPSS v.25 and EQS v.6.2. Specifically, SPSS was used for the descriptive results of the study and to calculate the reliability coefficient by means of the statistic Cronbach's alpha. Involvement in cyberbullying by countries was analyzed with crossed tables including chi squared and Cramer's V measure of association. Similarly, adjusted standardized residuals (ASR) were calculated to verify in what cells the differences were statistically significant. If ASR are higher than ±1.96, they indicate *p* < 0.05; if ASR values are higher than ±2.58, they show *p* < 0.01; and if values are higher than ±3.27, they show *p* < 0.001.

The EQS program was used to carry out the CFA of the instruments as well as to test the proposed structural equation model and the factorial invariance. The following indexes were used: Satorra-Bentler χ^2^; Comparative Fit Index (CFI); Non-normed Fit Index (NNFI); Root Mean Square Error of Approximation (RMSEA) with the confidence interval at 90%; Standardized Root Mean Square Residual (SRMR); and Akaike Information Criterion (AIC). Hu and Bentler [69] state that if the values of the indexes CFI and NNFI are higher than 0.95, this shows a good fit to the model. If the values are lower than 0.08, it is adequate for the measure SRMR. For the RMSEA index, values lower than 0.05 show a good fit, and values ranging from 0.05 and 0.08 show a reasonable fit [72]. For structural equation models (SEM), the Least Squares (LS) estimation method was used, assuming the variables were absolute and using polichoric matrixes. The calculated SEM present the coefficients of determination adjusted r squared (R^2^), the standardized coefficients beta (β) and their significance value (p) with an asterisk.

The Delta (Δ) differences between the fit indicators (NNFI, CFI and SRMR) were also considered to test the invariance degree. The cut-off point suggested in the literature to accept the hypothesis of invariance across groups is a change of 0.01 [73]. A multigroup analysis was performed using EQS V.6.2 too.

In addition, effect sizes (ƒ^2^ = 0.02–0.14 [small]; 0.15–0.34 [medium]; ≥ 0.35 [large]), statistical power (1- *β* ≥ 0.80) and confidence interval (CI = 95%) were analyzed for each individual trajectory in the SEM of each country. In the case of *R*^2^, the standard error (*SEE*) was also included. These analyses were carried out with the G-Power program.

For all the statistics, the confidence interval was at least 95%, *p* < 0.05.

## Results

### Prevalence of cyberbullying

The levels of prevalence of cyberbullying were higher in Ecuador in comparison with Spain (χ^2^ [3, 24943] = 763,030, *p* < 0.001; V-Cramer = 0.175, p < 0.001). The prevalence of cyberaggressors, and especially cybervictim-cyberaggressor, was higher in Ecuador than in Spain. Nevertheless, the levels of cybervictims did not differ between both countries (see Table 1). If all the roles of involvement, cybervictims, cyberaggressors and cybervictim-cyberaggressors are considered, it can be observed that in Spain, 16.8% of Secondary students were involved in cyberbullying behaviors while this percentage amounts to 28.1% in the case of Ecuador.

**Table 1:** Percentage of involvement in cyberbullying (CB).

		Spain (n = 14206)	Ecuador (n = 10737)
Role	Cybervictim	8.8%	8.7%
		ASR = .3	ASR = -.3
	Cyberaggressor	3.1%***	5.1%***
		ASR = -8.2	ASR = 8.2
	Cybervictim-Cyberaggressor	4.9%***	14.3%***
		ASR = -25.6	ASR = 25.6
	Not involved	83.2%***	71.9%***
		ASR = 21.4	ASR = -21.4

(*** p < 0.001), (ASR: Adjusted standardized residual).

### Predictive capacity of psychosocial variables on cyberaggression and cybervictimization

**Fig 1:**
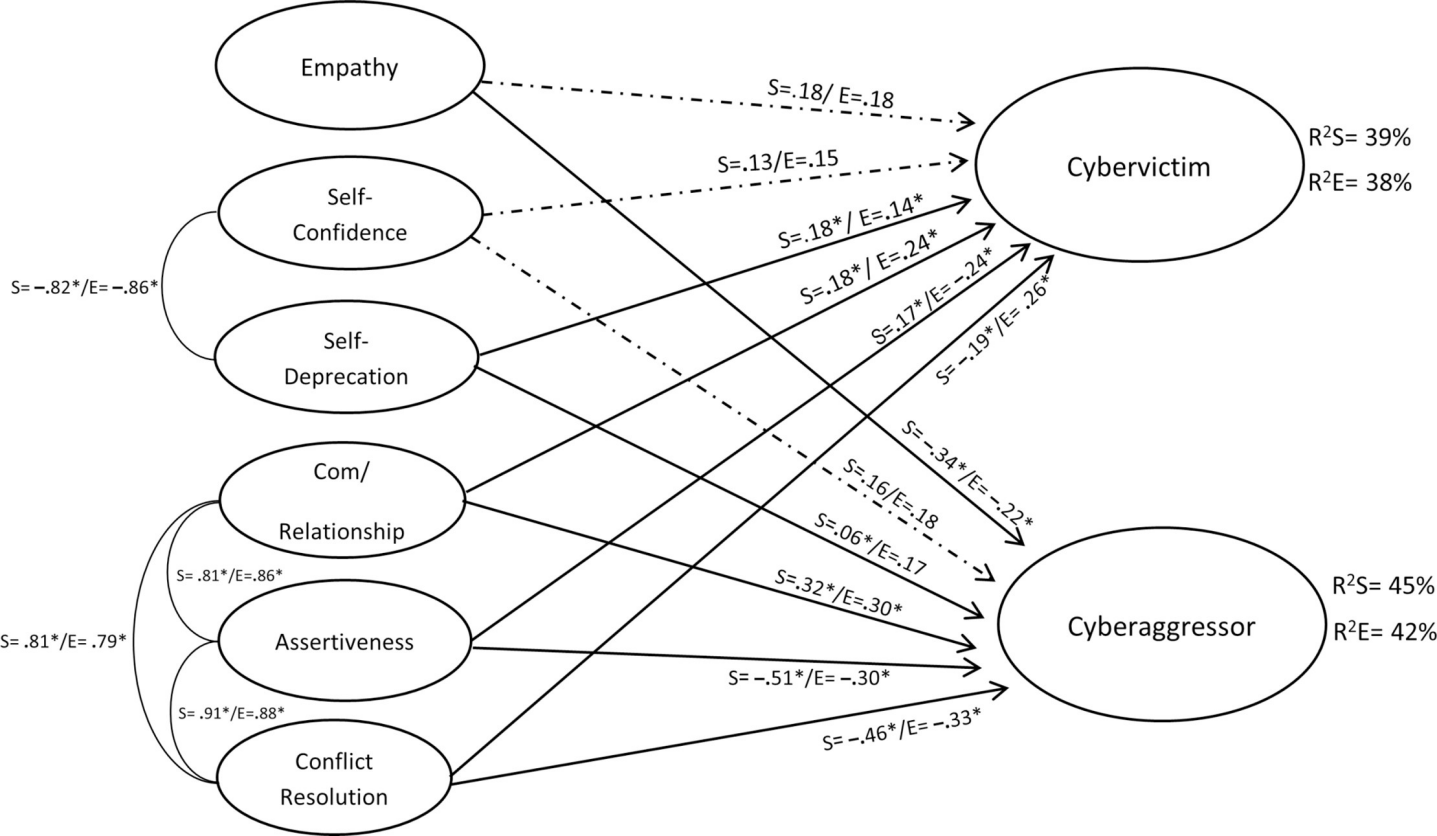
Model for the sample from Ecuador and Spain. E = Ecuador; S = Spain.

To address the second objective, an analysis was carried out with structural equation models to test the hypothesized theoretical model, according to which the psychological variables considered in this study would significantly be related and influenced, which would allow to predict involvement in certain behaviors of cyberaggression and cybervictimization. A model per country was carried out to know the differences or similarities between both. In the model with the Ecuadorian sample, optimal fit indexes were obtained (*χ*^2^_S-B_ = 4604.771; *χ*^2^_S-B_/(1298) = 3.547; *p* < 0.001; CFI = 0.983; NNFI = 0.982; RMSEA = 0.034 (90% CI [0.032, 0.036]); SRMR = 0.047; AIC = 4343.773), which explained 38% of variance for cybervictimization (*R*^*2*^ = 0.38; *SEE* = 0.577; *p* = 0.03; ƒ^2^ = 0.28 [medium]) and 42% for cyberaggression (*R*^*2*^ = 0.42; *SEE* = 0.475; *p* = 0.02; ƒ^2^ = 0.31 [medium]) (see Fig 1). In this model, high indexes of empathy predicted a lower involvement in cyberaggression; the same happened with assertiveness and conflict-resolution skills. Nevertheless, a high level of communicative and relational skills was associated with a higher level of cyberaggression. The other variables did not present any statistically significant incidence on cyberaggression. In the case of cybervictimization, high indexes of self-deprecation, high levels of communicative and relational skills, and high levels of conflict-resolution skills were associated with higher indexes of cybervictimization. Low levels of assertiveness were also linked to higher levels of cybervictimization.

In the Spanish sample, the model explained 39% of variance for cybervictimization (*R*^*2*^ = 0.39; *SEE* = 0.351; *p* = 0.03; ƒ^2^ = 0.21 [medium]) and 45% for cyberaggression (*R*^*2*^ = 0.45; *SEE* = 0.286; *p* = 0.02 ƒ^2^ = 0.37 [large]), (see Fig 1) with optimal fit indexes of the model (*χ*^2^_S-B_ = 5609.460; *χ*^2^_S-B_/(1298) = 4.321; *p* < 0.001; CFI = 0.972; NNFI = 0.973; RMSEA = 0.052 (90% CI [0.050, 0.056]); SRMR = 0.067; AIC = 5345.461) (see Fig 1). What is more, the effect of self-deprecation must be added to the association of variables present in the Ecuadorian sample in the case of Spain, although with modest values. The higher the levels of self-deprecation are, the higher the levels of cyberaggression will be. In the case of cybervictimization, the model between countries is very similar. The only difference can be found in assertiveness and conflict-resolution skills, which are related to cybervictimization in an opposite sense to the previous model, that is, the higher the level of assertiveness is, the higher the level of cybervictimization will be; the lower the level of conflict-resolution skills is, the lower the cybervictimization will be.

### Predictors of cyberaggression and cybervictimization: Spain *versus* Ecuador

The factorial invariance was calculated between the samples obtained in Ecuador and Spain to test if both samples could share a common theoretical model. Initially, the indexes were obtained with the total sample of both countries (*χ*^2^S-B = 5362.323; *χ*^2^S-B / (1298) = 4.131; p < .001; CFI = 0.973; NNFI = 0.974; RMSEA = 0.050 (90% CI [0.048, 0.052]); SRMR = 0.066; AIC = 5827.193), obtaining optimal adjustments (see Fig 1). Then the results showed the existence of a configural and metric invariance since the delta values (Δ) were below the cut-off point (see Table 2). Therefore, it can be suggested that the structure of the predictive-explanatory model of involvement for cyberbullying is similar in both countries.

**Table 2:** CFA by country and configuration and metric invariance.

	Country			Multigroup Analysis		
Mod	Spain	Ecuador	Unconstrained	Mod 1	Mod 2	Mod 3
***χ***^**2**^_**S-B**_	5609.460	4604.771	5003.501	5380.944	5690.461	5965.019
***df***	1298	1298	2618	2663	2716	2728
				(Δ*df = 45*)	(Δ*df = 98*)	(Δ*df = 110*)
***p***	0.001	0.001	0.001	0.003	0.013	0.017
**NNFI**	0.973	0.982	0.970	0.968	0.964	0.963
**CFI**	0.972	0.983	0.980	0.975	0.974	0.972
**RMSEA**	0.052	0.034	0.046	0.050	0.051	0.052
	[0.050, 0.056]	[0.032, 0.036]	[0.045, 0.046]	[0.049, 0.050]	[0.050, 0.052]	[0.050, 0.054]
**SRMR**	0.067	0.047	0.064	0.068	0.070	0.071
**AIC**	5345.461	4343.773	4376.177	5088.821	5175.601	5530.650
**Δ*χ***^**2**^_**S-B**_			--	377.443	686.960	961.518
				(n. s.)	(n. s.)	(n. s.)
**ΔNNFI**			--	0.002	0.006	0.007
**ΔCFI**			--	0.005	0.006	0.008
**ΔRMSEA**			--	0.004	0.005	0.006
**ΔSRMR**			--	0.004	0.006	0.007

Mod 1 = constrained soft; Mod 2 = constrained medium; Mod 3 = constrained hard; n.s. = not significant.

Likewise, the statistical powers and the size of the effects for each individual trajectory showed adequate values, all with high power (1-*β* > 0.80) and with mean effect values (*f*^2^ = 0.15–0.35) in both countries (see Table 3).

**Table 3:** Statistical power, effect size, and confidence interval for each individual trajectory.

Dependent Variable (Country)	Independent Variable	*β*	*p*	1- *β*	ƒ^2^	(CI 95%)	
						Lower limit	Upper limit
Cyber-victimization (Ecuador)	Empathy	0.18	**0.134**	--	--	--	--
	Self-Confidence	0.15	**0.234**	--	--	--	--
	Self-Deprecation	0.14	0.021	0.961	0.201	0.142	0.170
	Com/Relationship	0.24	0.040	0.901	0.120	0.107	0.115
	Assertiveness	-0.24	0.012	0.966	0.192	-0.130	-0.108
	Conflict Resolution	0.26	0.019	0.928	0.231	0.007	0.026
Cyber-victimization (Spain)	Empathy	0.18	**0.064**	--	--	--	--
	Self-Confidence	0.13	**0.191**	--	--	--	--
	Self-Deprecation	0.18	0.035	0.972	0.308	0.154	0.162
	Com/Relationship	0.18	0.030	0.952	0.221	0.183	0.197
	Assertiveness	0.17	0.022	0.988	0.197	0.083	0.105
	Conflict Resolution	-0.19	0.020	0.952	0.291	-0.042	-0.021
Cyber-aggression (Ecuador)	Empathy	-0.22	0.037	0.928	0.231	-0.144	-0.121
	Self-Confidence	0.18	**0.531**	--	--	--	--
	Self-Deprecation	0.17	**0.481**	--	--	--	--
	Com/Relationship	0.30	0.029	0.992	0.112	0.111	0.133
	Assertiveness	-0.30	0.012	0.951	0.120	-0.126	-0.102
	Conflict Resolution	-0.33	0.047	0.901	0.192	-0.201	-0.166
Cyber-aggression (Spain)	Empathy	-0.34	0.044	0.955	0.226	-0.221	-0.182
	Self-Confidence	0.16	**0.301**	--	--	--	--
	Self-Deprecation	0.06	0.021	0.904	0.188	0.043	0.055
	Com/Relationship	0.32	0.028	0.919	0.201	0.187	0.205
	Assertiveness	-0.51	0.045	0.973	0.272	-0.381	-0.327
	Conflict Resolution	-0.46	0.011	0.942	0.220	-0.292	-0.260

β = Standardized beta coefficient; *p* = significance (≤0.05); 1- β = statistical power; ƒ^2^ = effect size.

## Discussion

In Spain, nearly 2 out of every 10 adolescents are directly involved in cyberbullying. In Ecuador, the situation is even more worrying as nearly 3 out of every 10 adolescents are involved in cyberbullying. These levels of involvement are significantly higher than the findings observed by Lee et al. in the USA [26]. However, the levels observed in the present study are within the ranges of involvement described in a recent systematic review of studies conducted in the USA [27]. In Spain, the level of involvement reported in the present study is, in comparison with other previous studies: lower than 3 out of every 10 students observed by other studies [28, 29]; and consistent with the results obtained by Herrera-López, Casas, et al. [33]. In Ecuador, the level of involvement detected in the present study is significantly higher to the one detected in its neighbor country Colombia [33].

In Spain and Ecuador, 1 out of every 10 adolescents recognizes him/herself as a cybervictim. This prevalence of cybervictims doubles the one observed by Lee et al. [26] and Chen et al. [17] with other different instruments. However, it is consistent with the prevalence of cybervictimization observed in Spain in different studies [28, 33, 34], carried out with the same instruments.

It is concluded that 3 out of every 100 adolescents recognize themselves as cyberaggressors in Spain, while in Ecuador the figure amounts to 5 out of every 100. The prevalence of cyberaggression in Spain is consistent with the one shown in previous studies in the USA [26] and in Spain [28, 33, 34]. The prevalence of cyberaggressors in Ecuador is equivalent to the one shown in Spain and higher to the one in Colombia [34].

Additionally, 5 out of every 100 people recognize themselves as cybervictims-cyberaggressors in Spain, while in Ecuador the figure amounts to 14 out of every 100. The prevalence of cybervictims-cyberaggressors in Spain is consistent with the one observed in studies carried out by Lee et al. in the USA [26] and in Spain [28, 33, 34]. Nevertheless, the prevalence of cybervictims-cyberaggressors in Ecuador is much higher than the one observed in studies carried out in different countries [28, 33, 34].

As we have noted, the rates of involvement in cyberbullying in Ecuador, especially in cyberaggression and cybervictim-cyberaggression, are higher than in Spain. This may be due to the fact that the rate of violence in Ecuador, if we measure it in terms of homicides, is much higher than that the one of Spain [74]. This situation can explain this difference in terms of another type of interpersonal violence such as cyberbullying.

As noted above, cyberbullying rates in Ecuador are higher than in neighboring Colombia. This is especially worrisome given that Colombia has higher levels of violence, in general terms, than Ecuador [74]. The fact that in South American countries the prevalence rates of cyberbullying are higher than in Europe or North America may be due to the normalization of violence that occurs in the most disadvantaged countries. This normalization in the society may be reflected in educational centers. In the same way, a shortage of scientifically valid programs has been detected in the South American continent [75], which probably affects this high prevalence. Self-esteem was expected to be negatively associated with cybervictimization [40, 44, 45]. This was the case not only in the Spanish adolescents but also in the Ecuadorian ones: self-deprecation was positively associated with cybervictimization. Positive self-esteem (or self-confidence) did not show an association with cybervictimization. This makes think that emotional aspects linked to a negative self-worth are those more connected to the risk of being a cybervictim.

Based on Brewer & Kerslake's study [44] on nearly 100 British adolescents, the hypothesis was that empathy will be associated with cybervictimization. Based on the conclusions drawn in Zych et al.'s review [55], it was expected that empathy would be positively associated with cybervictimization. Nevertheless, empathy did not show an association with cybervictimization either for Spanish or Ecuadorian adolescents. This may be due to the fact that in the present study we have considered empathy as a unifactorial construct, while in the Zych et al., reviews it was specified that it was affective empathy and not general empathy (cognitive empathy and affective empathy) that would be related to cybervictimization [55]. When considering empathy as a whole, it has not shown an association with cybervictimization as indicated by the authors [55].

Some authors have revealed the existence of an association between social skills and cybervictimization [66, 67]. This relation was observed in both Spanish and Ecuadorian adolescents. Communicative and relational social skills were positively associated with cybervictimization in both countries. However, assertiveness was positively associated with cybervictimization in Spain and negatively associated with it in Ecuador. Conflict-resolution skills were negatively associated with cybervictimization in Spain and positively in Ecuador. The transnational differences detected, regarding the prediction of cyber-aggression based on assertiveness and the ability to resolve conflicts, could have an explanation based on cultural and contextual differences. It is possible that students who feel they have more conflict resolution skills and are less assertive, in the relationships between schoolchildren in Ecuador -with rates of interpersonal violence much higher than those in Spain- have a greater chance of coming into conflict with other classmates. This could condition that they are more likely to suffer cybervictimization. On the other hand, in the relationships between schoolchildren in Spain -with lower levels of interpersonal violence and better school climate–it could be interpreted that being more assertive but with less conflict resolution skills could influence some students to have more confrontations and being the target of cybervictimization. The predictive ability of social skills observed on wide samples of adolescents in Spain and Ecuador contrast with the observation in a previous study in Spain that used the same instruments for each one of the controlled ethnic-cultural groups [7], where social skills did not show a predictive capacity of cybervictimization.

Self-esteem was expected to be negatively associated with cyberaggression [44, 45]. This hypothesis is only confirmed in adolescents from Spain: negative self-esteem (or self-deprecation) is associated with cyberaggression. However, this hypothesis was not confirmed in Ecuador. Perhaps, in more violent societies, such as Ecuador, the fact of being a cyberbully would not be so associated with psychological variables as with context variables. But this hypothesis should be contrasted with specific studies.

Considering previous studies, empathy was expected to be negatively associated with cyberaggression [44, 52]. This hypothesis is confirmed in both Spanish and Ecuadorian adolescents. In fact, this is consistent with the conclusion of Zych et al.'s metanalysis [55]: high levels of cyberaggression are related to low levels of empathy.

Social skills were expected to show an association with cyberaggression. This hypothesis is confirmed in the samples of adolescents from Spain and Ecuador. Regarding each of the social skills studied in the present work, the different countries show equivalent predictive patterns. Cyberaggression could be: positively associated by means of communicative and relational skills; and negatively associated by means of assertiveness and conflict-resolution skills. The predictive capacity of communicative and relational skills and assertiveness is consistent with the previous observation of another study carried out in Spain only on some ethnic-cultural subgroups [7].

There are cultural similarities between Spain and Ecuador due to the language and reciprocal historical influence. However, there are great differences regarding their socio-economic level, ethnic-cultural diversity, access and availability of technology and internet, parenting styles and education, among others. It is possible that these differences have an influence on the differences observed between countries both in the roles of involvement and in the predictors of cyberbullying (e.g., assertiveness and conflict resolution skills predict cybervictimization in the opposite direction in Spain and in Ecuador).

### Strengths, limitations, and future lines of research

The present study has some limitations but also allows to move forward into the development of some new lines of research. The transversal methodology by means of self-reports, despite fitting the objectives and hypothesis under study, presents some limitations as well. Due to the methodology used (cross-sectional study), causality cannot be inferred so the reason why the relationships occur is unknown. If being involved in cyberbullying influences psychosocial variables or by having certain psychosocial variables, one is more likely to become involved in this phenomenon, either in cyberaggression or cybervictimization. Another limitation of the study is the lack of participation/rejection rates due to the large volume of participating centers. It would have been interesting to know the reasons for the refusal to participate in the study, in this way it could be known if there has been any repetitive error in the sample. For example, centers with higher rates of cyberbullying refuse to participate due to the fear that their results will be known.

Although a common data collection protocol was established in both countries, the fact of collecting data in Spain online and in Ecuador on paper may have influenced the results. Perhaps using a paper support can make participants more task-focused and respond more accurately. These possible differences should be tested in the future.

Despite these limitations, our study combines a large sample obtained in two countries with robust analyzes that allow us to improve our knowledge of cyberbullying and its psychosocial predictors. For future research, data collection could combine self-reports and hetero-reports and triangulate the results regarding involvement in cyberbullying through different roles. It could be interesting to carry out longitudinal studies on this matter. This would make possible to know about the evolution of cyberbullying and move from the prediction to the deepening in knowledge of protective factors and precursors of this phenomenon. It would be convenient that future studies on cyberbullying would consider not only the transcultural comparison between different countries but also the ethnic-cultural comparison within each country [7].

It would also be convenient to include in future studies the registry of variables related to parenting and educational styles, as well as access and availability of technology and the Internet, since they could show a relationship or explain some of the possible transnational differences. In the same way, it would be interesting to explore whether there were differences between genders or ages. By obtaining this information we will be able to be more precise when it comes to knowing the variables that most influence cyberbullying and develop better programs for its prevention.

Another possible line of work could investigate whether in different types of cyber-aggression and cybervictimization these SEM models would be maintained or would change substantially. Cyberbullying does not manifest itself in a single way, but it can have different faces. Knowing all of them in depth will give us a better knowledge.

## Conclusions

Our findings show that the incidence of cyberbullying in Ecuador is higher than in Spain. Moreover, self-deprecation, communicative or relational skills, assertiveness and conflict-resolution skills were significantly associated with cybervictimization. These psychosocial variables together with empathy were significantly associated with cyberaggression. From the conclusions regarding the prevalence of cyberbullying, it would be convenient to develop specific educational programs of prevention both in Secondary Education and at the final stage of Primary Education. This could contribute to anticipate its emergence. From the conclusions regarding the prediction of cyberbullying, it can be inferred that there are several aspects that such preventive educational programs should include. The prediction patterns of cyberaggression between countries are very consistent. This makes possible to create a prevention proposal of cyberaggression from a transcultural point of view. The proposal would be based on socioemotional support and education in order to: a) dissolve and avoid negative self-esteem; b) stimulate the development of affective and cognitive empathy; and c) promote assertive behavior and conflict-resolution skills. However, the prediction patterns of cybervictimization between countries are less consistent. Taking similarities into account, the prevention proposal of cybervictimization of transcultural nature could consider the socioemotional support and education in order to: a) manage and overcome negative self-worth; and b) successfully develop communicative/relational social skills, assertiveness and conflict-resolution skills.

Universal prevention and intervention proposals could be more effective if they could be adapted and specified considering cultural aspects of cultural groups that could be helpful. The differences in the access to cyberspace, technological development, customs, habits, and cultural values, among other aspects, could condition the degree of involvement and the weight of protective factors and precursors of cyberbullying.
